# Antiviral Oseltamivir Is not Removed or Degraded in Normal Sewage Water Treatment: Implications for Development of Resistance by Influenza A Virus

**DOI:** 10.1371/journal.pone.0000986

**Published:** 2007-10-03

**Authors:** Jerker Fick, Richard H. Lindberg, Mats Tysklind, Paul D. Haemig, Jonas Waldenström, Anders Wallensten, Björn Olsen

**Affiliations:** 1 Department of Chemistry, Umeå University, Umeå, Sweden; 2 Section for Zoonotic Ecology and Epidemiology, Kalmar University, Kalmar, Sweden; 3 Department of Animal Ecology, Lund University, Lund, Sweden; 4 Smedby Health Center, Kalmar County Council, Kalmar, Sweden; 5 Division of Virology, Department of Molecular and Clinical Medicine (IMK) Faculty of Health Sciences, Linköping University, Linköping, Sweden; 6 Section of Infectious Diseases, Department of Clinical Sciences, Uppsala University Hospital, Uppsala, Sweden; University of Cambridge, United Kingdom

## Abstract

Oseltamivir is the main antiviral for treatment and prevention of pandemic influenza. The increase in oseltamivir resistance reported recently has therefore sparked a debate on how to use oseltamivir in non pandemic influenza and the risks associated with wide spread use during a pandemic. Several questions have been asked about the fate of oseltamivir in the sewage treatment plants and in the environment. We have assessed the fate of oseltamivir and discuss the implications of environmental residues of oseltamivir regarding the occurrence of resistance. A series of batch experiments that simulated normal sewage treatment with oseltamivir present was conducted and the UV-spectra of oseltamivir were recorded. Findings: Our experiments show that the active moiety of oseltamivir is not removed in normal sewage water treatments and is not degraded substantially by UV light radiation, and that the active substance is released in waste water leaving the plant. Our conclusion is that a ubiquitous use of oseltamivir may result in selection pressures in the environment that favor development of drug-resistance.

## Introduction

**Figure 1:**
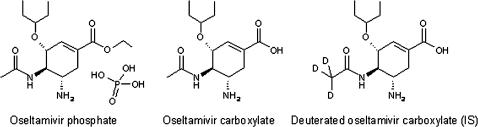
Oseltamivir phosphate (OP), oseltamivir carboxylate (OC), the internal standard (IS) used, and deuterated oseltamivir carboxylate (OC_D3_).

Influenza A virus is a zoonotic pathogen with a large environmental reservoir in anatids, especially dabbling ducks [1], which also infects a number of mammals, including pigs, horses, seals and canines [2]. Over the past centuries, the virus has been transmitted to humans on several occasions, causing flu pandemics and seasonal epidemic influenzas [3]. At present, there are only few antiviral compounds available to treat human influenza. The most important, oseltamivir, or oseltamivir phosphate (OP), is a prodrug that is extensively metabolized (>75%) in the human liver to oseltamivir carboxylate (OC), the active moiety (Figure 1) [4]. OC is not metabolized further and is excreted unchanged [4]. Oseltamivir is widely used for treatment of seasonal flu and is considered an important first-line defense in the event of a future influenza pandemic [5], [6]. This compound is a neuraminidase inhibitor, which mimics the natural sialic acid substrate and binds to the active site, preventing the viral neuraminidase protein from cleaving host-cell receptors, thereby interfering with the release of new virus particles from infected cells [5]. To investigate whether or not oseltamivir is removed in normal sewage water treatment, we set up and ran batch experiments that simulated normal sewage treatment with oseltamivir present. In these experiments, we used OC, since this is the active moiety and also the molecule excreted by patients.

A conventional sewage treatment plant functions in three steps: (1) mechanical treatment, (2) chemical treatment and (3) biological (activated sludge) treatment. In the mechanical treatment phase, raw sewage water passes through a grid that first removes large objects, then lipids and sand. Chemical treatment subsequently reduces nutrients, such as phosphorus, in the aqueous phase by addition of FeCl_3_ or FeSO_4_, while biological treatment reduces organic content. The sludge produced is removed in the clarifiers following each step and treated further with different techniques. The treated water is then released and diluted into receiving water courses.

## Methods

### Experimental design

In our experiments we used three different water solutions, each representing one of the three phases in the conventional sewage treatment process outlined in the previous paragraph: (1) raw sewage water, (2) water from combined mechanical/chemical treatment, and (3) water from activated sludge treatment. All three water solutions were collected in one liter bottles as grab samples during two days in June 2006 at Umeå Sewage Treatment Plant, (for a detailed description of the plant, see references [7], [8]. During the two days of collecting water, normal conditions were reported for water treatment, with some minor rainfall during the second day. To avoid misinterpretation of the results, quantification of OC in all the raw sewage water samples was made and additional batch experiments (3 ½ h) conducted using tap water to assess possible OC degradation or adsorption to glass walls.

All batch experiments started within 1 hour of water collection and were conducted as follows: 200 ng of OC, was added to 200 mL of each type of water and gently stirred in an open 500 mL flask at 20°C. The duration of each experiment was determined by the hydraulic retention time in the plant: (1) raw sewage water, 2 ½ h (the assumed time the water spends in the sewage line upon reaching the sewage treatment plant); (2) water from mechanical/chemical treatment, 1 ½ h; and (3) water from activated sludge treatment, 3 ½ h. This approach has been used previously with relevant and reproducible results [9]. When the hydraulic retention time was reached, the sample was immediately subjected to solid phase extraction (SPE) and liquid chromatography electrospray tandem mass spectrometry (LC-ESI-MS/MS). Each experiment was conducted in triplicate for each water solution type to assess day-to-day variation of the sewage water treatment process and experiments: (day 1) n = 1; and (day 2) n = 2. The amount of suspended solids (SS) and pH of the various water samples included in the batch experiments can be seen in Table 1.

**Table 1:** pH and suspended solids (SS) of the water included in the batch experiments

	pH		SS	
			mg/L	
	day 1	day 2	day 1	day 2
RSW^a^	8,05	7,60	290	170
mech/chem^b^	7,66	7,47	510	570
AST^c^	7,65	7,31	2700	2800

a Raw sewage water.

b Water from mechanical/chemical treatment.

c Water from activated sludge treatment

### Chemicals

Oseltamivir carboxylate (OC), (RO0640802-002; lot: 01007B243804) and Oseltamivir carboxylate labelled with deuterium (OC_D3_), (RO0604802-004; lot: 511-001-2197/4) were obtained from Roche (F. Hoffmann-La Roche Ltd, Basel, Switzerland). Formic acid, ammonium hydroxide 25% and methanol (HPLC-grade) were purchased from JT Baker (Deventer, the Netherlands), acetonitrile (HPLC-grade) from Fischer Chemicals (Zurich, Switzerland) and sulphuric acid from Merck (Darmstadt, Germany). The purified water (resistivity, 18.2 MΩ cm) was prepared by an ELGA MAXIMA HPLC ultra pure water system (ELGA, High Wycombe Bucks, England), equipped with a UV radiation source. Buffer solutions pH 5 (citric acid/sodium hydroxid) and pH 9 (boric acid/potassium chloride/sodium hydroxide) were bought from Merck (Darmstadt, Germany) and were of ceripure grade. Buffer solution pH 7 (phosphate) was purchased from JT Baker (Deventer, the Netherlands) and was of “Baker Analyzed” grade. Standard stock solutions of OC and OC_D3_, 100 ng mL^−1^, were prepared in water (10 mL) and kept at 4 °C.

### Solid phase extraction and quantification of OC in sewage water

Sewage water samples were filtered through 0.45 µm MF™-membrane filters (Millipore, Sundbyberg, Sweden) before acidification to pH 3 using sulphuric acid. This low pH ensured a high recovery of the amphoteric OC. 700 ng of the deuterated internal standard OC_D3_ was added to each sample and 200 mL aliquot of each sample was withdrawn and subjected to extraction. The Strata-X-C (200 mg, 6 mL) mixed mode cation exchange sorbent (Phenomenex, email: internationalphenomenex.com) used for the solid phase extraction (SPE) was conditioned and equilibrated by 2.0 ml of methanol and 2.0 ml of deionized water. The samples were applied to the SPE columns at a flow rate of 5 mL min^−1^. Impurities were removed by 2.0 ml 0.1% sulphuric acid and the sorbents dried with air during 1 min at 10” Hg. Supposed neutral and acidic components were removed by 2 mL of methanol and wasted, followed by elution of the analytes by 2 mL of 5% NH_4_OH in methanol collected with 10 ml glass vials. The eluates were evaporated to approximately 20 µl using air and then reconstituted in acetonitrile in water (1∶1), containing 0.1% formic acid, to a final extract volume of 1.0 ml. Quantification of OC was performed by internal standard calibration by comparison of area ratios OC/OC_D3_ in sample extracts and calibration solution.

### Liquid chromatography electrospray tandem mass spectrometry

A 10 µl aliquot of sample extracts and calibration solutions was injected into a YMC Hydrosphere C18 analytical column, 150×4.6 mm i.d., 5 µm particle size, (YMC Inc., Wilmington, NC, US) following a 10×4 mm i.d., 5 µm particle size, YMC Hydrosphere C18 guard column using an AS 3000 autoinjector (Thermo Finnigan, San Jose, CA, US). OC and OC_D3_ were chromatographically separated during 5 min by 50% H_2_0 balanced with acetonitrile, both containing 0.1% (v/v) formic acid, at a flow rate of 0.8 ml min^−1^ generated by a P4000 HPLC pump (Spectra system, Thermo Finnigan) at 25°C.

An LCQ Duo ion trap mass spectrometer (Thermo Finnigan) was used together with an electrospray ion source in positive ion mode. The source voltage was maintained at a constant 6.0 kV and the heated capillary temperature set to 250°C. The MS/MS parameters were optimised semi-automatically for the analytes using LCQ Duo internal software whilst the collision energy, to produce daughter ions, was manually optimised.

For OC and OC_D3_ parent and daughter ion *m/z*, collision energy, retention time, extraction yields in tap water and raw sewage water and LOQ, see Table 2.

**Table 2:** LC-ESI-MS/MS parameters and results of the method validation.

	PI^a^	CE^b^	DI^c^	t_r_	Extraction yield %				LOQ
	m/z	%	m/z	min	10 mL^d^	100 mL^d^	200 mL^e^	500 mL^d^	ng/L
OC	284.9	21	196.9	1.97	114/107	97/100	90 (10)	107/91	15
OC_D3_	287.9	20	199.9	1.96	-	-	-	-	-

a Parent ion.

b Collision energy, arbitrary unit.

c Daughter ion.

d Raw sewage water/tap water.

e Cumulative mean and RSD in % (in parenthesis) of three SPEs and three injections, respectively, on LC-ESI-MS/MS.

The *m/z* obtained for the OC and OC_D3_ parent and daughter ions are consistent with those reported by Wiltshere et al. [10] who also identified that the resultant daughter ion (M^+^ -88 amu) had lost the pentyloxy sidechain. The retention time of the deuterated intermal standard, OC_D3_, was analogous to OC, and the linearity of the calibration curve was above 0.99. Memory effects during LC-ESI-MS/MS were not observed. The extraction yields OC with the mixed mode cation exchange sorbent were in most cases close to 100%, regardless of matrix subjected to extraction, and the precision was acceptable with RSD below 21%. Breakthrough effects were not observed at any level of sample load volume used, and sequential elutions (3×2 mL) with MeOH or NH_4_OH did not contain OC above LOQ. Extraction yields of the internal standard OC_D3_ were not determined but assumed to be analogous to OC due to their similarity in physico/chemical properties.

### Method validation

Extraction yields of OC were assessed by fortification experiments. 1000 ng of OC was added to the following matrices prior to extraction with the method presented above (in mL): tap water-10, 100 and 500; and raw sewage water (filtered through 0.45 µm)-10, 100, 200 (n = 3) and 500. OC_D3_, 700 ng, were added to the reconstituted extracts (1 mL during these experiments) prior to injection on the LC-ESI-MS/MS. The extraction yields were evaluated by comparison of LC-ESI-MS/MS peak area ratios of OC/OC_D3_ in sample extracts against a calibrate solution of 1000 ng mL^−1^ of OC in 1 mL acetonitrile in water (1∶1), 0.1% formic acid. Evaluation of matrix effects during LC-ESI-MS/MS was assessed by comparison of the OC_D3_ peak area in chromatograms of the calibrate solution and the sample extracts of the following degree of enrichment during SPE: 0, 10, 100, 200, and 500 times.

An internal standard calibration curve of eight levels, 1–1000 ng mL^−1^, of OC was injected into the LC-ESI-MS/MS. The limit of quantification (LOQ) was evaluated by using ten times the signal to noise ratio of OC, 1000 ng mL^−1^ in sample extract (enriched 200 times).

Blank samples of tap water and raw sewage water subjected to SPE and acetonitrile in water (1∶1), containing 0.1% formic acid, were regularly injected into the LC-ESI-MS/MS to control and reduce potential memory effects.

### Ultraviolet absorption spectra

Standard solutions of OC (10^−3^ M, 284,35 mg l^−1^) were prepared in buffer solutions with pH 5, 7 and 9. UV-spectra were recorded on a UV-VIS-NIR scanning spectrophotometer (UV-3101PC, Shimadzu), which was set to scan over 250–800 nm. To correct for differences in cell performance, a baseline correction was made with corresponding buffer solution in both sample and reference cells.

## Results

No OC was detected in the raw sewage water and no losses observed in the batch experiment using tap water, which minimizes the possibilities of positive or negative sampling artefacts. OC and OC_D3_ were readily affected by matrix components in the raw sewage water (Figure S1). However, the combination of a high recovery of OC during solid phase extraction of sewage water, and the use of a deuterated internal standard with almost identical physico-chemical properties, makes the developed analytical methodology very suitable for environmental monitoring in various aqueous matrices.

**Figure 2:**
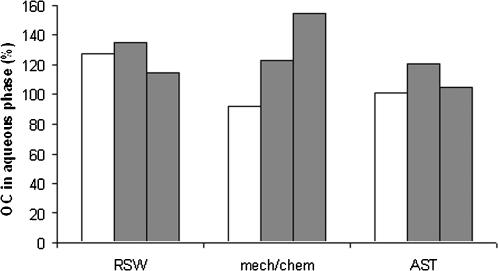
Results of batch experiments to assess removal of oseltamivir carboxylate (OC) from the aqueous phase during conventional sewage water treatment. Shown is the OC remaining in the aqueous phase after the batch experiments. Abbreviations: RSW, raw sewage water; mech/chem., mechanical and chemical treatment of sewage water; and AST, activated sludge treatment of sewage water. White and grey bars represent day 1 and day 2 of the batch experiments, respectively.

Removal of OC due to degradation and/or sorption to sludge was not observed in the batch experiments. The day-to-day variation in terms of batch experiment and treatment process seemed to be minor, and all results are close to a 100% recovery of the added OC in the aqueous phase (Figure 2). Recoveries were 107% (S.D. 19) in the raw sewage water, 126% (S.D. 8) in the water from combined mechanical/chemical treatment, and 125% (S.D. 27) in the water from activated sludge treatment.

These findings suggest that, since OC is not removed during sewage treatment, it will enter local aquatic environments in areas where oseltamivir is prescribed to patients for therapeutic use.

A way to estimate the levels in the aquatic environment is to calculate the highest predicted environmental concentration (PEC) according to,
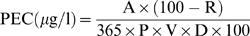
where A is the total actual pharmaceutical sales (µg year^−1^), R the removal rate due to loss by adsorption to sludge particles, volatilization, hydrolysis or biodegradation (%), P the human population (number of individuals), V the volume of wastewater per capita per day (l day^−1^) and D a factor for dilution of waste water by surface water flow.

The country where oseltamivir is used most is Japan [11], Roche estimates that 6 million of the 16 million Japanese individuals infected by an influenza virus during the influenza season 2004/2005 received oseltamivir [12]. Environmental concentrations should therefore be the highest in Japan, with the calculated PEC value equal to PEC_surfacewater_Japan_ = 0.028 µg l^−1^ where A = 2650 kg (Estimated volume during flu season 2004/2005)^12^ (Estimation is based on 30% pediatric dosage, 70% adult dosage and OP converted to OC), R = 0 (No removal), P = 127 417 244 (2005), V = 200 (Default) and D = 10 (Default). This calculation provides a national annual average and does not consider local factors such as catchment size, population density or flow rates. Another factor not included is seasonal consumption, such as increased usage during flu season. This PEC level can be compared to the IC_50_ (concentration that causes 50% inhibition) of OC, which depends heavily on type of virus and exposure system, but such low levels as 0.28–0.81 nM (IC_50_) have been reported [11], [13]. This corresponds to a concentration of 0.08–0.23 µg L^−1^, i.e. a concentration of the same magnitude as the calculated PEC value. Singer et al. [14] estimated the environmental levels of OC during treatment and prevention of a pandemic influenza in Europe and North America. Estimations showed that environmental levels would differ significantly between different catchments and maximum concentrations would range from<0,3 µg L^−1^ to 32 µg L^−1^. Predicted levels in Japan during the flu season are thus comparable to predicted levels in some catchment areas during treatment and prevention of a pandemic.

Incidentally, Japan also has high rate of emerging resistance to oseltamivir [15]. Kiso et al. [16] reported that 18% (9 patients) of the influenza A virus in children had mutations that made them 300–10^5^ times more resistant to oseltamivir.

**Figure 3:**
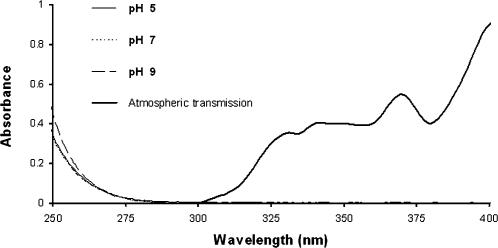
UV-spectra of oseltamivir carboxylate, at pH 5, 7, 9 and atmospheric transmission at sea level (expressed on a scale of 0 to 1).

Once a pharmaceutical enters the aquatic environment, photochemical degradation represents another possible degradation pathway [17]. However, the UV-spectra of OC show no absorbance in the interval 295–700 nm, which excludes direct aqueous photolysis as a major degradation pathway. Only chemicals that are able to adsorb solar radiation can be degraded and solar radiation in wavelengths shorter than 290–300 nm does not reach the Earth's surface, see Figure 3, and radiation with wavelengths>700 nm do not contain enough energy to break bonds within the molecules.

Pharmaceuticals in the aquatic environment are primarily removed by three degradation pathways: sorption, biodegradation and photolysis. Our experimental results show that OC is not readily degraded by any of these three, which implies that OC is released into aquatic environments at varying concentrations and is not readily removed. Thus, ducks, poultry and humans, or their excretions carrying influenza viruses, may encounter water contaminated with oseltamivir. Under these circumstances, where influenza viruses come in contact with low concentrations of the drug, the stage is set for the evolution of oseltamivir-resistant influenza strains [1].

## Discussion

The problem of environmental contamination becomes even more serious when one considers the ecology of influenza A virus. The life cycle of influenza A virus is intrinsically linked to water where they can remain active for extended periods; and under cold conditions for months [18]–[20]. Most subtypes circulate in wild ducks [1] that become infected by contaminated water establish infection in the gastrointestinal tract, where the virus multiplies and is excreted in large numbers via the faeces [2], [21]. Thus, both the infection and the pharmacological effect of OC occur in the gastrointestinal tract. Due to the poor bioavailability of OC [4] it is therefore theoretically possible that exposed ducks have OC at levels close to the IC_50_ in the gastrointestinal tract and that this could promote a selection process towards drug-resistance. Singer and coworkers recently stated this was a potential risk during treatment and prevention of a pandemic influenza [14], and our calculations imply that this can also be a potential risk in Japan today.

In some localities, wild ducks, domesticated ducks, poultry and humans all live in close proximity, transmit influenza viruses to each other, and conceivably ingest low concentrations of OC in treated or untreated sewage water. The water outside a sewage plant may comprise a particularly high risk microhabitat where ducks carrying a multitude of influenza virus strains encounter low levels of oseltamivir. This is particularly so because large numbers of ducks often gather in the warm nutrient-rich waters leaving sewage treatment plants, especially in cold climates where this warmer water remains ice-free year-round.

In some parts of the world, chicken manure is used as fertilizer in fish farming, a practice that can increase the spread of avian influenza [22]. During an outbreak of avian influenza in poultry, this activity could expose ducks, and other animals frequenting fish ponds downstream from sewage outlets, to highly pathogenic influenza virus strains from the chickens and low concentrations of OC from sewage water

Studies have shown that most oseltamivir resistant strains detected so far, have been detected in patients not treated with oseltamivir [11], [23]. It remains to be seen if such resistant strains are transmitted from treated individuals, the result of natural variation in the absence of oseltamivir altogether, or due to the selective pressure of low doses of oseltamivir in the environment. Previous research has, however, shown that it is quite easy for influenza A virus to develop resistance to oseltamivir [24], [25]. For example, a single amino acid substitution, from histidine to tyrosine at position 274 (N2 numbering system; N2 numbering is used throughout this article) of the neuraminidase gene “converted” an oseltamivir sensitive H5N1 influenza A virus into a resistant strain, with about a 400–600-fold higher resistance to OC [25]. Most resistant influenza A virus have mutations in the neuraminidase gene leading to amino acid substitutions predominantly at positions 119, 152, 274 and 292 of the enzyme's active site [5]. All the resistant variants thus far have contained specific mutations in the neuraminidase molecule; but since neuraminidase serves an essential purpose, mutations that allow the virus to “survive” must not inactivate the enzyme [15]. Carr et al. [26] showed, for example, that mutations at position 292 compromised viral fitness to such extent that it was considered to have no clinical consequences. However, an experimental study in ferrets [27] has shown that mutations at position 119 do not compromise viral fitness. The authors state that “if such viruses are transmitted, it is uncertain whether, over time, they could predominate over susceptible strains” [27].

In conclusion, our experimental results, theoretical calculations and hypothesis imply the possibility that ubiquitous use of oseltamivir may result in selection pressures in the environment that favor development of drug-resistance. This raises the all-important question as to whether or not such a risk should be taken, or if a more restricted use of these agents should be advocated? This is an opinion shared by other researchers [28], and we would like to add that the effects of pharmaceuticals continuously released into the environment should not be underestimated and certainly investigated carefully before widespread use of a drug is encouraged.

## Supporting Information

Figure S1 Peak area of OCD3 in calibrate solution and in raw sewage water (RSW) extracts, as a function of enrichment 10, 100, 200, and, 500 times during SPE.
(0.54 MB TIF)
