# Supplementary figures and images for: Antiviral Oseltamivir Is not Removed or Degraded in Normal Sewage Water Treatment: Implications for Development of Resistance by Influenza A Virus
Source: PLoS One. 2007 Oct 3;2(10):e986. doi: 10.1371/journal.pone.0000986 (PMC1991587; doi:10.1371/journal.pone.0000986)

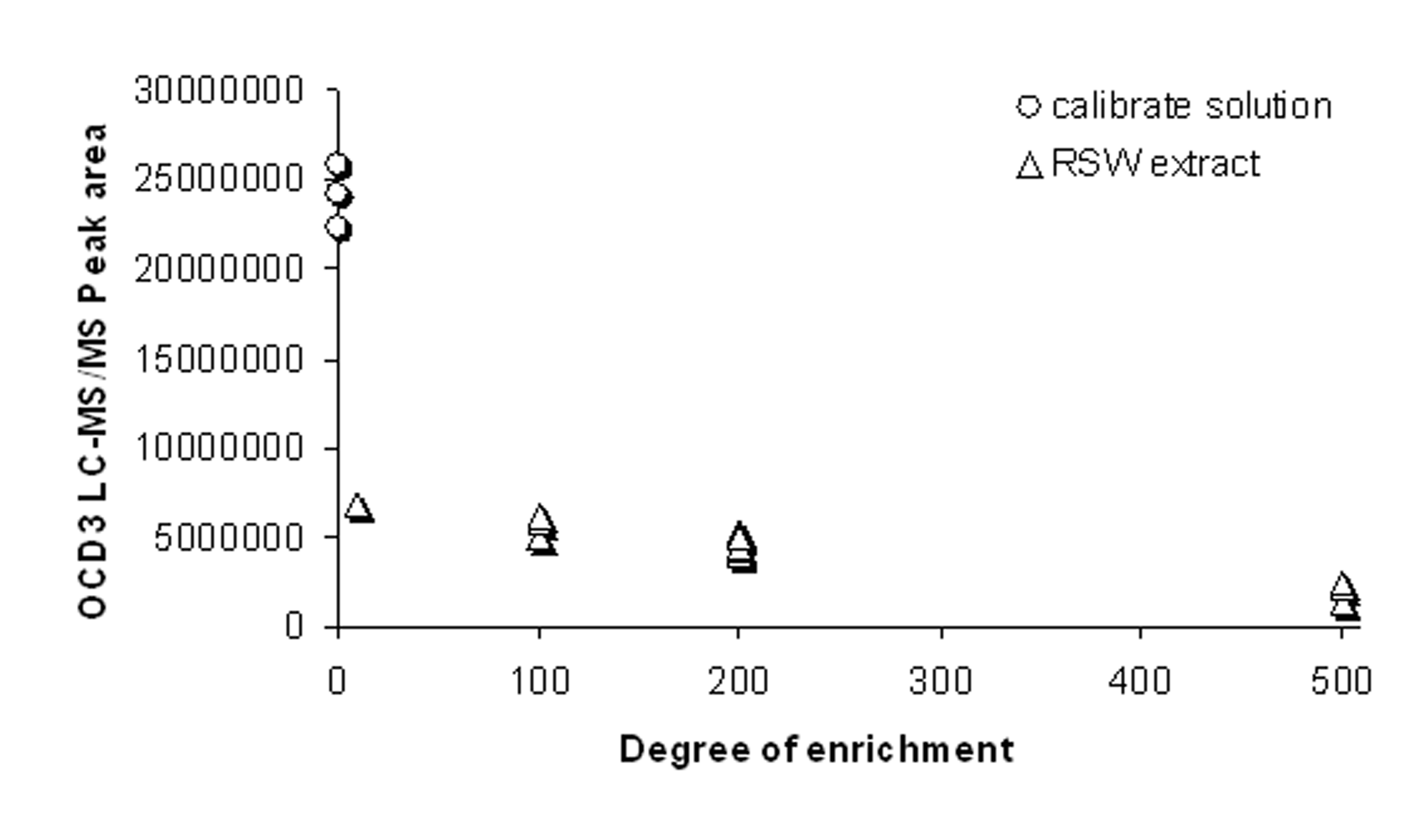

Supplement: Figure S1 — Peak area of OCD3 in calibrate solution and in raw sewage water (RSW) extracts, as a function of enrichment 10, 100, 200, and, 500 times during SPE. (0.54 MB TIF) [file pone.0000986.s001.tif]
